# The effects of ingestion of hormonal host factors on the longevity and insecticide resistance phenotype of the major malaria vector *Anopheles arabiensis* (Diptera: Culicidae)

**DOI:** 10.1371/journal.pone.0180909

**Published:** 2017-07-11

**Authors:** Shüné V. Oliver, Basil D. Brooke

**Affiliations:** 1 Centre for Emerging, Zoonotic and Parasitic Diseases, National Institute for Communicable Diseases, Johannesburg, South Africa; 2 Wits Research Institute for Malaria, School of Pathology, Faculty of Health Sciences, University of the Witwatersrand, Johannesburg, South Africa; Institute of Plant Physiology and Ecology Shanghai Institutes for Biological Sciences, CHINA

## Abstract

Exogenous vertebrate-derived factors circulating in the blood have the capacity to modulate the biology of haematophagous insects. These include insulin, insulin growth factor 1 (IGF) and transforming growth factor β1 (TGFβ). The effects of the consumption of these three proteins were examined on laboratory strains of *Anopheles arabiensis*. SENN, an insecticide susceptible strain and SENN DDT, a resistant strain selected from SENN, were fed with host factor-supplemented sucrose. Adult longevity was measured and insecticide resistance phenotype over time was assessed by WHO bioassay. Detoxification and oxidative stress defence enzyme activity was assessed calorimetrically. Insulin supplementation augmented insecticide resistance in young adult mosquitoes. This effect was due to the hormonal nature of the protein, as heat-denatured insulin did not elicit the same response. In contrast, IGF and TGFβ consumption generally reduced the expression of insecticide resistance. Insulin ingestion significantly reduced longevity in the insecticide susceptible strain. IGF elicited the same response in the susceptible strain, while TGF consumption had no effect on either strain. Consumption of all factors significantly decreased Glutathione S-transferase activity and increased cytochrome P450 and superoxide dismutase activity. This suggests that the altered detoxification phenotype is mediated primarily by cytochrome P450 activity, which would result in an increase in oxidative stress. The increased superoxide dismutase activity suggests that this enzyme class alleviates the oxidative stress as opposed to glutathione-based redox systems. Oxidative stress responses play a crucial role in insecticide resistance and longevity. These data show that ingested hormonal factors can affect mosquito longevity and insecticide susceptibility, both of which are important characteristics in terms of malaria transmission and control.

## Introduction

Haematophagy by mosquitoes that is necessary for embryonic development and enables disease transmission can also affect the life history parameters of adult females. For example, blood is potentially toxic to mosquitoes due to haemoglobin derived iron (reviewed in [1]). Furthermore, for anthropogenic mosquitoes, circulating blood-borne components ingested by the human host, such as antibiotics, have life history consequences for adult female mosquitoes [2]. More importantly, endogenous circulating host factors also have the capacity to alter the biology of vector mosquitoes.

One of the most important blood-borne factors ingested by mosquitoes is insulin. Exogenous host-derived insulin can exert numerous effects on the mosquito. These include stimulation of ecdysteroid production [3] and the immunological response to *Plasmodium* infection [4, 5]. This is due to the capacity of insulin to activate invertebrate receptors of insulin-like proteins (ILPs). These proteins activate a conserved insulin/insulin-like growth factor pathway that regulates immunity, reproduction, growth and longevity in both vertebrates and invertebrates (reviewed in [6]). Insulin is also a significant vertebrate host factor for consideration as hyperinsulinaemia often results from malaria parasite infection and subsequent quinine therapy [7, 8]. Therefore, malaria vectors feeding on malaria-infected persons may be exposed to even higher dosages of insulin, inducing various effects. For example, laboratory studies have demonstrated that high levels of insulin supplementation increase parasite load [4] and decrease the lifespan of the malaria vector *Anopheles stephensi* [9]. Therefore, the consumption of substantial amounts of exogenous insulin is not advantageous to female mosquitoes.

Insulin growth factor 1 (IGF) activates the same receptors as insulin, but results in a very different biological response in both humans and insect disease vectors. In contrast to insulin, IGF-1 levels fall precipitously during a malaria episode, correlating well with the host’s increased parasitaemia [10]. In further contrast to the response to insulin, ingested human IGF appears to advantage *An*. *stephensi* by increasing lifespan and augmenting the vector’s immune response to *Plasmodium falciparum* [10].

Cytokines are circulating vertebrate signalling proteins involved in the regulation of host immune responses. Similar to the insulin-like receptors, invertebrates have also been found to possess cytokine-like factors, and this suggests that exogenous cytokines may also be able to regulate aspects of vector biology [11].The transforming growth factor (TGF)-β family of is a group of cytokines, with TGF-β1 exerting numerous pleiotropic immunological effects. These include regulation of the responses that limit parasite growth [12, 13]. Ingested human TGF-β1 is activated in the mosquito midgut by components required by the female to digest blood [14]. Like insulin and IGF, the existence of conserved TGF-β signalling pathways and proteins suggest that these pathways can be activated by exogenous human TGF-β.

Although the effects of these vertebrate-derived host factors have been examined for various mosquito life traits of epidemiological importance, the potential interplay of these factors and insecticide resistance has never been examined. Furthermore, the effects of these factors on longevity have only been examined in insecticide susceptible individuals. It has previously been demonstrated that longevity in insecticide resistant mosquitoes differs from their susceptible counterparts [15, 16]. Therefore, when examining the effects of longevity modulators, it is important to consider both insecticide resistant and susceptible individuals.

Malaria in South Africa is primarily controlled by indoor spraying of residual insecticides (IRS) [17]. Although this method has proved successful over several decades, low-level transmission continues and is likely caused by residual populations of the major malaria vector *An*. *arabiensis* [18, 19]. This species is less susceptible to the IRS programmes because infective females often feed and rest outdoors, effectively out of reach of insecticide deposits [20, 21]. In addition, populations of this species, especially in South Africa’s northern KwaZulu-Natal province, have developed resistance to several classes of insecticide [22]. As residual malaria is a threat to South Africa’s malaria elimination agenda, quantifying the effects of those factors that affect epidemiologically significant traits such as longevity and insecticide resistance is important.

This study aimed to examine the effects of insulin, IGF and TGF consumption on the longevity and expression of insecticide resistance in two laboratory strains of *An*. *arabiensis* which differ in resistance phenotype.

## Materials and methods

### Materials

Two laboratory reared *An*. *arabiensis* strains were used in this study. The SENN strain was colonised from Sennar, Sudan in 1980. From this strain, the SENN DDT strain was selected. This strain is routinely exposed to 4% DDT. Although this strain was selected on DDT, it currently displays resistance to DDT, deltamethrin, permethrin, λ-cyhalothrin and malathion [15]. Currently, mortality levels for SENN DDT average 15% for DDT, 18% for permethrin, 35% for deltamethrin, 40% for λ-cyhalothrin and 28% for malathion. This resistance appears to be mediated by elevated P450, GST and general esterase activity. This strain is also fixed for the L1014F *kdr* mutation. A recent study has also implicated elevated oxidative stress enzyme activity in the resistance phenotype [16, 23]. All strains were housed in the Botha de Meillon insectary in Johannesburg, South Africa, at 25^°^C (±2^°^C) and 80% humidity (±5%). Mosquitoes were reared as per [24].

## Methods

### Effect of host factor ingestion on adult female longevity

In this study, both the SENN and SENN DDT strains were assayed. As only females would encounter host factors in their lifetime, the effect of host factor ingestion was only examined on females. For each experiment, 30 females were used for each dietary treatment. Three replicates were performed for adults arising from 3 different egg batches (cohorts). The females were allowed access to 10% sucrose supplemented with 3.4x10^-4^μM recombinant human insulin (Sigma Aldrich: I2643) reconstituted in 25mM.litre^-1^ HEPES buffer pH 8.2, with an untreated control constituted of 10% sucrose supplemented with 25mM.litre^-1^ HEPES buffer pH 8.2, as per [9]For the IGF treatment, females were exposed to either a low dose of IGF (0.013μM reconstituted in 10mM HCl) or a high dose of IGF (0.133μM reconstituted in 10mM HCl) as per [25]. An untreated control consisted of 10% sucrose supplemented with 10mM HCl. For the TGF treatment a low dose of 2000ppm or a high dose of 10 000ppm TGF reconstituted in 10mM citric acid was used. A sucrose solution supplemented with 10mM citric acid served as an untreated control. The females used in this experiment were not allowed to mate or have access to blood during their lifetimes. Mortality was recorded daily, with cadavers removed on a daily basis until all adults were dead. Survival was analysed using the Kaplan-Meier estimator with the log-rank test used as a measure of significance.

To determine whether any effects seen by insulin were due to its hormonal activity, a separate set of cohorts, as described above, were supplied with sucrose supplemented with heat denatured human insulin of the same concentration.

Superoxide dismutase mimetics have been demonstrated to have variable effects on longevity [26, 27]. The manganese superoxide dismutase mimetic Manganese III tetrakis (4-benzoic acid porphyrin (MnTBAP) was shown to increase longevity in *An*. *stephensi* [9]. Therefore, cohorts as described above were supplied with 10% sucrose supplemented with 0.05mMol.litre^-1^ MnTBAP dissolved in 25mM HEPES buffer pH 8.2.

For all sugar treatments (controls), the sugar was changed on a daily basis.

### Effect of insulin on insecticide resistance phenotype

The effect of insulin supplementation on insecticide resistance phenotype with age was examined. Non-blood fed, unmated SENN DDT females were supplied with either 10% sucrose supplemented with 3.4x10^-4^μM active recombinant human insulin in 25mM.litre^-1^ HEPES buffer pH 8.2, sucrose supplemented with heat denatured human insulin at the same concentration, or an unsupplemented sucrose solution. Sugar waters were changed on a daily basis, and adults were drawn from these treatments at the ages of 3, 7, 11, 15, 18 and 21 days and exposed to 4% DDT using standard WHO bioassays[28]. Mortality was recorded after 24 hours. A control exposed to untreated paper as well as a completely unexposed environmental control was used with every DDT exposure assay.

SENN DDT females supplied with either active insulin supplemented sucrose or unsupplemented sucrose were also drawn from their respective cohorts at the ages of 3, 7, 11,15, 18 and 21 days and were exposed to either 0.05% deltamethrin, 1.5% permethrin, or 5% malathion using standard WHO bioassays[28], with controls as described for the active/denatured insulin assay. Mortality was scored after 24 hours. For all insulin assays, exposed mosquitoes were not returned to the experimental cohort, and all insecticide exposed mosquitoes only underwent a single insecticide dose. Malathion bioassays were only performed until 15 days of age, as 100% mortality was reached at this age.

### Effects of IGF and TGF on insecticide resistance phenotype

Newly emerged, unmated, non-blood fed SENN DDT females were supplied with either 10% sucrose supplemented with 0.013μM IGF (low dose IGF), 2000ppm TGF (low dose TGF) or untreated 10% sucrose. At the ages of 3, 7, 11 and 15 days, samples were drawn from each treatment group and exposed to either 4% DDT, 0.05% deltamethrin, 0.05% λ-cyhalothrin or 5% malathion using standard WHO bioassays [28], with controls as described for the insulin bioassays. As with the insulin bioassays, all insecticide exposed individuals received only a single treatment, and were not returned to experimental cohorts.

### Dietary supplementation and detoxification enzyme activity

Newly emerged SENN and SENN DDT females were placed into cages where they were supplied with either unsupplemented 10% sucrose, 10% sucrose supplemented with 3.4x10^-4^μM active recombinant human insulin, 0.013μM IGF or 2000ppm TGF. These treatments were their only dietary sources for 3 days. Sugar treatments were changed daily, and females were not allowed to blood feed or mate.

At the age of 3 days, after constant supplementation with a host factor, 96 females were drawn from each treatment, cold terminated and homogenised in PCR grade water. Protein content was quantified using the Bradford method [29]. Haeme peroxidase activity, Glutathione S-transferase (GST) activity and α and β-esterase activity were determined as per [30], [31, 32]), respectively.

### Dietary supplementation and oxidative stress enzyme activity

The samples used to determine oxidative stress enzyme activity were prepared in the same manner as those used for detoxification enzyme analysis. Catalase and Glutathione peroxidase activity was determined as described in [16]. Superoxide dismutase activity was determined using a kit assay based on the production of WST-1 formazan (Sigma Aldrich: 19160).

## Results

### Insulin supplementation and longevity

**Fig 1:**
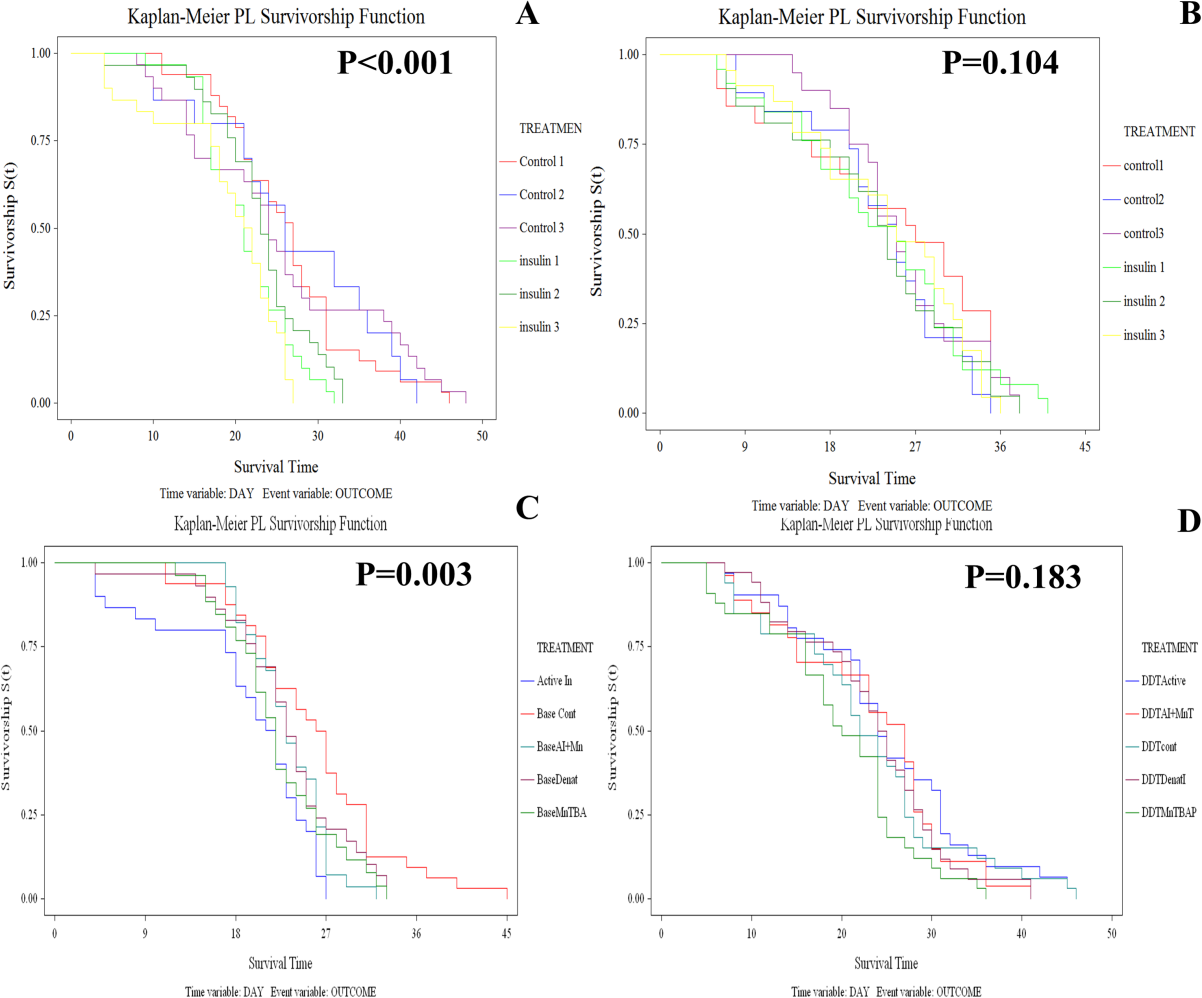
The effects of insulin dietary supplementation on the longevity of the *An*. *arabiensis* strains SENN and SENN DDT. When provided with *ad libitum* access to sucrose supplemented with insulin, the insecticide susceptible SENN strain showed a significant decrease in longevity (A), but this was not observed in the resistant SENN DDT strain (B). For the susceptible SENN strain it was demonstrated that active insulin (blue line) significantly decreased longevity compared to untreated individuals (red line), while denatured insulin (purple line) did not. For this strain, the SOD-mimetic MnTBAP (green line) also significantly decreased longevity (C). In contrast, none of the dietary treatments affected the longevity of the resistant SENN strain (D).

The supplementation of diet with active recombinant human insulin significantly reduced the longevity of the SENN strain (Log rank test: p<0.01; χ^2^ = 11.27) (Fig 1A), but not SENN DDT (Log rank test: p = 0.9; χ^2^ = 6.72) (Fig 1B). Although active insulin significantly reduced longevity in SENN, denatured insulin did not induce a significant change in longevity (Log rank test: p = 0.1; χ^2^ = 2.82). The MnSOD mimetic MnTBAP alone significantly reduced longevity (Log rank test: p = 0.04; χ^2^ = 4.27), as did the combination of MnTBAP and insulin (Log rank test: p = 0.03; χ^2^ = 5.01)(Fig 1C). As insulin had no effect on the longevity of SENN DDT, none of the other treatments (denatured insulin, MnTBAP, MnTBAP+insulin) had any significant effects on SENN DDT (Log rank test: p = 0.9; χ^2^ = 4.52) (Fig 1D).

### Growth factor supplementation and longevity

**Fig 2:**
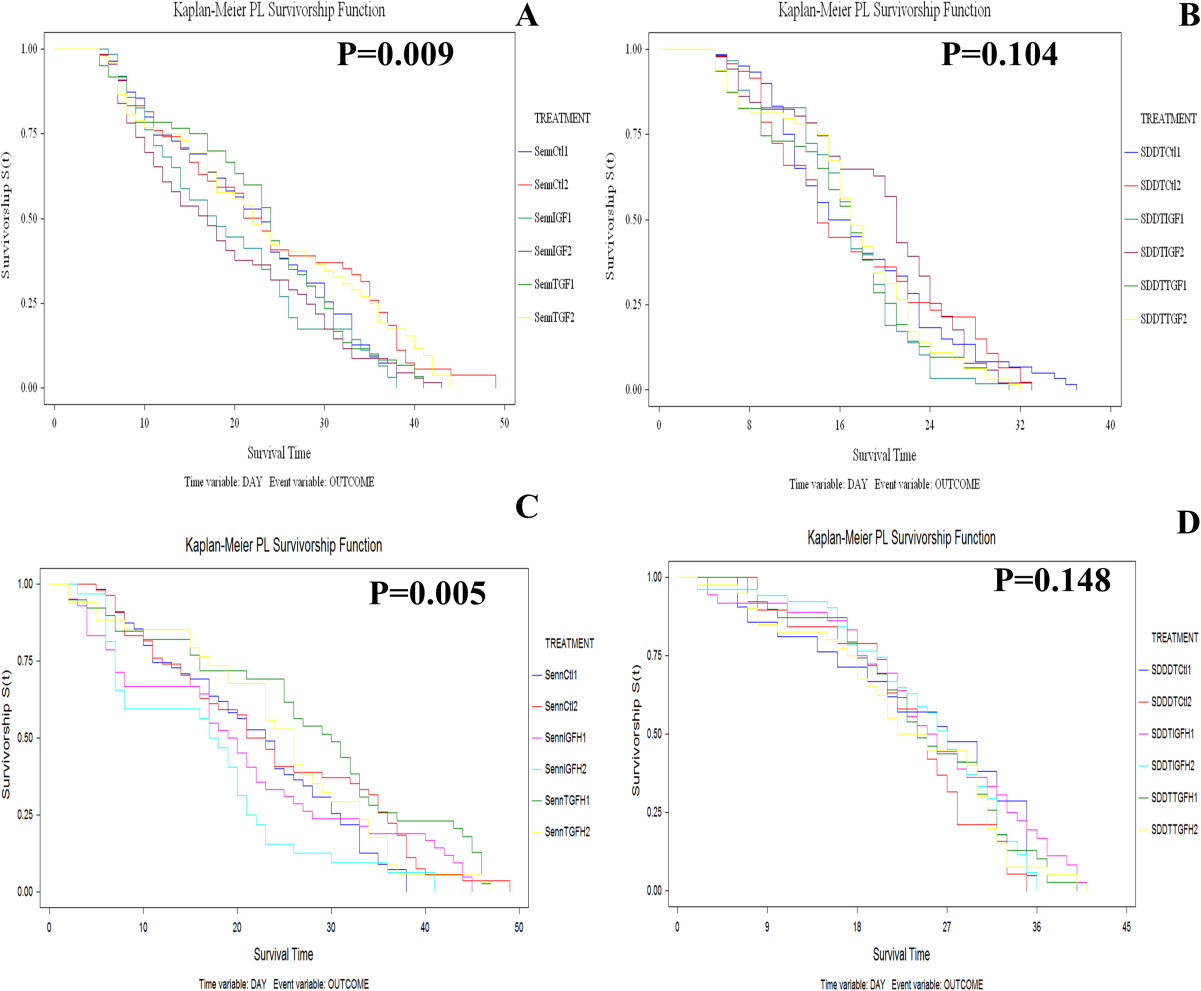
The effects of growth factor and cytokine dietary supplementation on the longevity of the *An*. *arabiensis* strains SENN and SENN DDT. When provided with *ad libitum* access to sucrose supplemented with low dose IGF, the insecticide susceptible SENN strain showed a significant decrease in longevity, while TGF had no effect (A). Neither of the low dose treatments had an effect on the longevity of the resistant SENN DDT strain (B). High dose IGF and TGF did not induce a significant change in longevity in the SENN strain, but the TGF treated individuals lived significantly longer (C). The high dosages did not alter the longevity of the SENN DDT strain. P-values indicate the significance after a log-rank test on all the treatments.

Dietary supplementation with low dose growth factors significantly reduced SENN longevity (Log rank test: p = 0.01; χ^2^ = 15.32) (Fig 2A). Although low dose TGF did not significantly reduce longevity (Log rank test: p = 0.84; χ^2^ = 4.84), low dose IGF significantly reduced longevity compared to the control (Log rank test: p = 0.01; χ^2^ = 11.13) as well as the TGF treatment (Log rank test: p = 0.01; χ^2^ = 10.85). Neither of the low dose treatments significantly affected longevity in the SENN DDT strain (Log rank test: p = 0.1; χ^2^ = 9.11) (Fig 2B). For the high dosages in the SENN strain, IGF had no significant effect on longevity (Log rank test: p = 0.2.75; χ^2^ = 3.87), and neither did TGF (Log rank test: p = 0.61; χ^2^ = 1.83) compared to the control. Individuals treated with high dose TGF lived significantly longer than individuals treated with high dose IGF (Log rank test: p = 0.002; χ^2^ = 14.5) (Fig 2C). As with low dose treatments, high dose treatments did not have any effect on SENN DDT longevity (Log rank test: p = 0.58; χ^2^ = 3.87) (Fig 2D).

### Insulin supplementation and insecticide resistance phenotype

**Fig 3:**
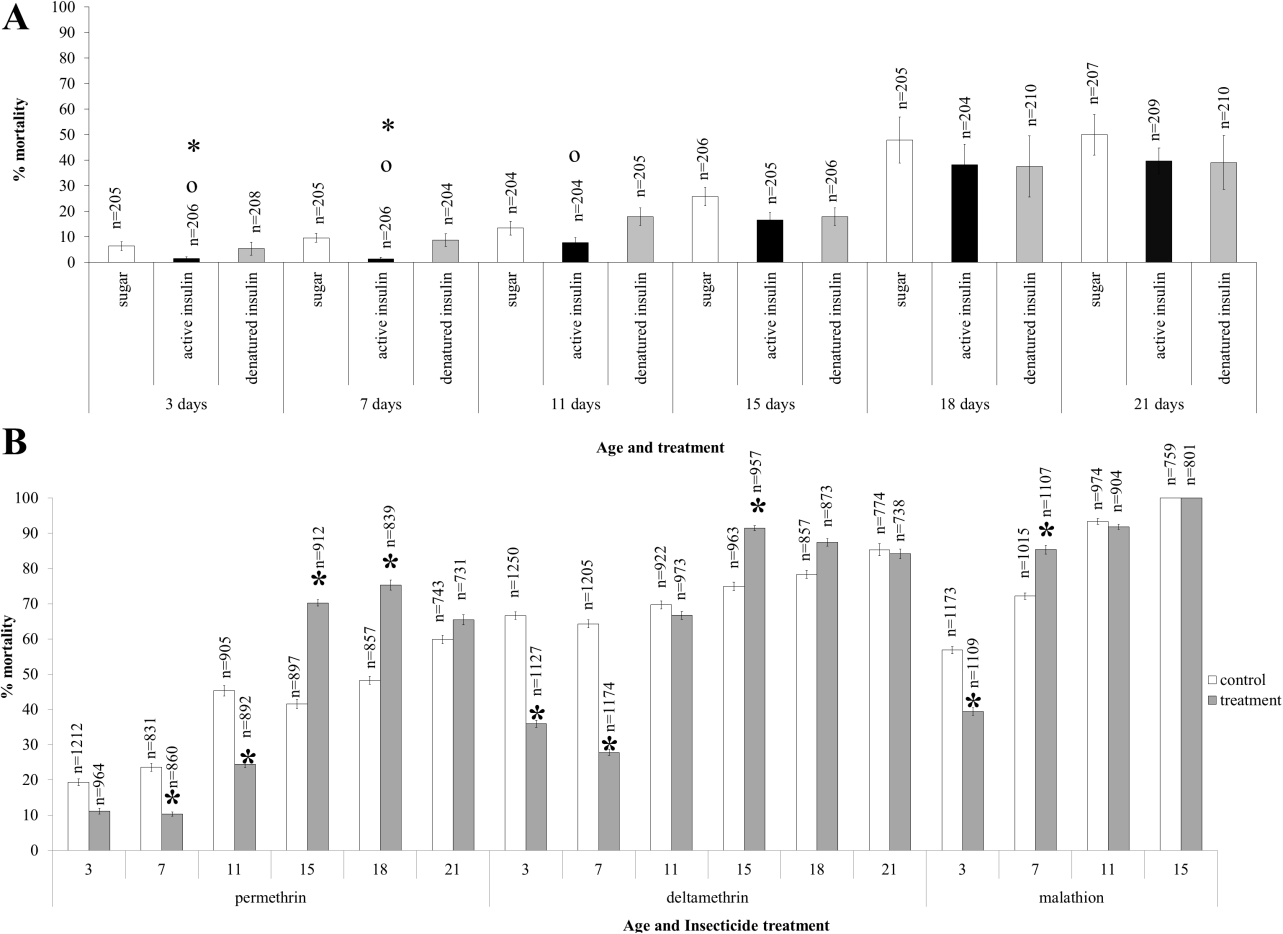
The effects of insulin dietary supplementation on the insecticide resistance phenotype of the insecticide resistant *An*. *arabiensis* strain SENN DDT. Active insulin significantly decreased DDT-induced mortality in the 3 and 7-day old mosquitoes, thereafter, the effect was lost. This was only observed when the mosquitoes were fed active insulin, as this effect was not observed when they were fed denatured insulin. Significant differences between active insulin and controls are indicated by an asterisk (*), while significant differences between active and denatured insulin are indicated by a circle (o) (A). A similar pattern was observed for other classes of insecticide. Insecticide-induced mortality was reduced in 7 and 11-day olds for permethrin, 3 and 7 day olds for deltamethrin and 3 day olds for malathion. Insulin treatment resulted in higher insecticide induced mortality at 15 and 18 days for permethrin, 15 days for deltamethin, and 7 days for malathion. Significant differences between treatment and control are indicated by an asterisk (*) (B).

The administration of active recombinant human insulin to SENN DDT females significantly reduced DDT induced mortality at the ages of 3 and 7 days (1-way ANOVA: p = 0.02; F = 4.39, df = 1), while denatured insulin at the same age did not have the same effect (2-sample t-test: p = 0.09; t = 1.68). Until the age of 11 days, ingestion of active insulin resulted in significantly lower DDT induced mortality than denatured insulin (1-way ANOVA: p<0.01; F = 13.4; df = 1). This effect ceased from the age of 15 days, where no significant differences in mortality existed between active and denatured insulin-treated groups and sugar fed controls (Generalised linear model: p = 0.64; t = -0.46) (Fig 3A).

The administration of active insulin to SENN DDT females over 15 days had a variable effect on the resistance phenotypes measured by exposures to permethrin, deltamethrin, and malathion. For deltamethrin and malathion, active insulin significantly reduced insecticide induced mortality in 3 day old adults (2-sample t-test: deltamethrin: p<0.01; t = -5.57; malathion: p<0.01; t = -3.14). Although deltamethrin-induced mortality was reduced at the age of 7 days (2-sample t-test: p = 0.04; t = -2.09), malathion-induced mortality was increased by insulin supplementation (2-sample t-test: p = 0.04; t = -2.09). Insulin supplementation decreased permethrin-induced mortality in 7-day old females (2-sample t-test: p<0.01; t = 3.25) and in 11 day old females (2-sample t-test: p = 0.03; t = 2.34). Insulin supplementation increased pyrethroid induced mortality in older females similar to malathion. Deltamethrin-induced mortality was increased at the age of 15 days (2-sample t-test: p = 0.01; t = 2.78), while permethrin-induced mortality was also increased at the age of 15 days (2-sample t-test: p<0.001; t = 4.22) and at 18 days (2-sample t-test: p<0.01; t = 4.33) (Fig 3B).

### Growth factors and insecticide resistance

**Fig 4:**
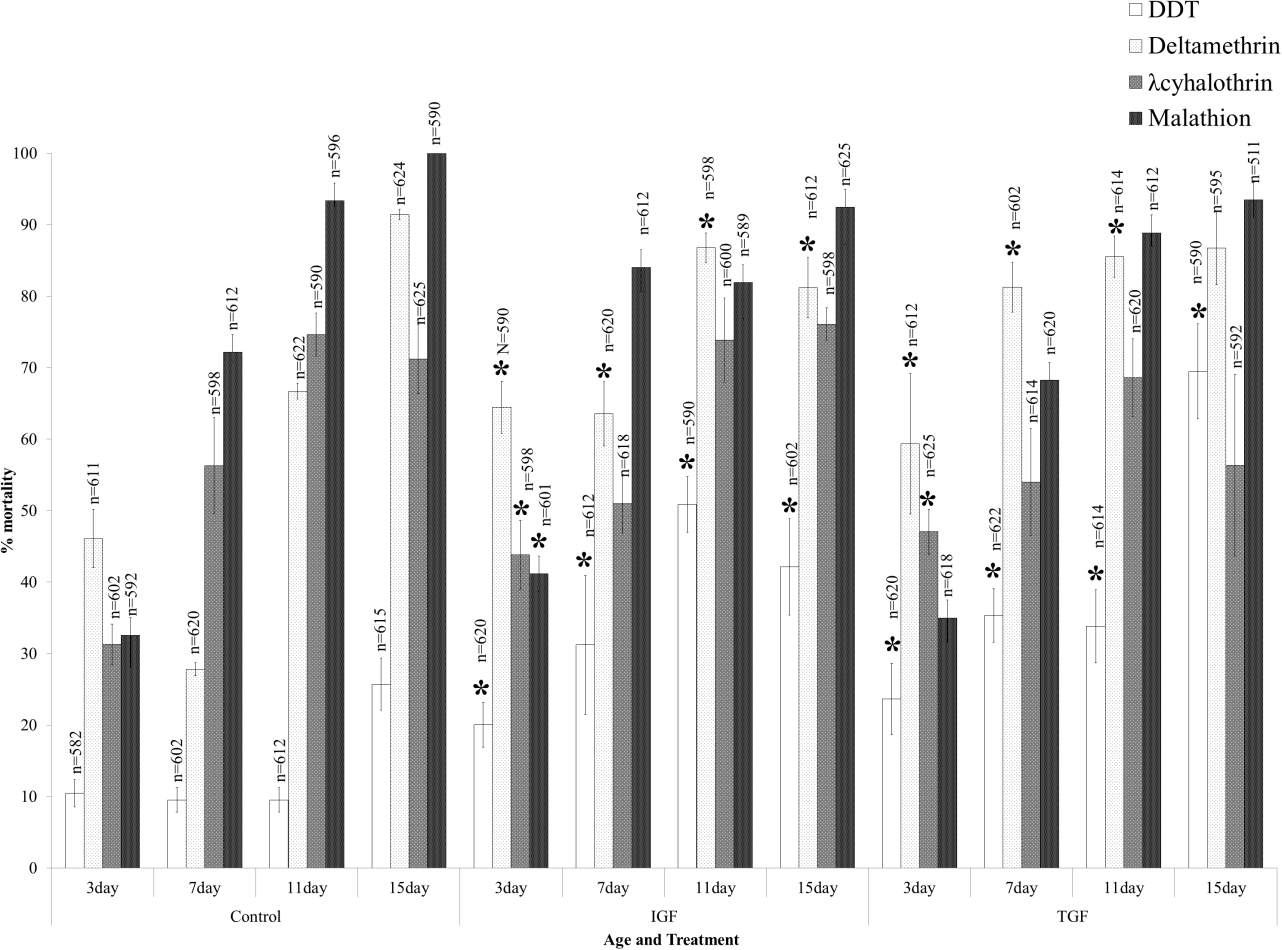
The effects of growth factor and cytokine dietary supplementation on the insecticide resistance phenotype of the insecticide resistant *An*. *arabiensis* strain SENN DDT. Consumption of sugar supplemented with IGF or TGF typically increased insecticide-induced mortality. This was particularly marked in 3-day old mosquitoes where insecticide-induced mortality increased for all insecticide classes. IGF consumption increased DDT-induced mortality at all ages, as well as deltamethrin-induced mortality. TGF consumption did not affect malathion-induced mortality at any age, but the 3 day olds were most susceptible to dietary induced increases in insecticide-induced mortality. DDT-induced mortality was increased by TGF consumption at all ages, while deltamethrin-induced mortality was only increased until 11 days of age. Significant differences from the control are indicated by an asterisk (*).

Dietary supplementation with IGF and TGF generally resulted in an increase in insecticide-induced mortality (Fig 4). This was particularly marked for IGF where, at 3 days of age, insecticide-induced mortality was significantly decreased for all four insecticides (2-sample t-test: DDT: p = 0.04, t = -2.18; deltamethrin: p<0.01, t = -3.35; λ-cyhalothrin: p = 0.03, t = 2.27; malathion: p = 0.04, t = -2.14). Similarly, at the age of 3 days TGF consumption significantly increased mortality induced by insecticide (2-sample t-test: DDT: p = 0.03, t = -2.38; deltamethrin: p = 0.03, t = 2.31; λ-cyhalothrin: p<0.01, t = 3.31) but not malathion (2-sample t-test: p = 0.62; t = -0.51). DDT-induced mortality was significantly increased by IGF consumption from 3–15 days of age (1-way ANOVA: p<0.01, F = 10.2, df = 3). Similarly, TGF consumption from 3–11 days increased DDT-induced mortality (1-way ANOVA: p<0.01, F = 21.6, df = 2), but not 15 days (2-sample t-test: p = 0.22; t = -1.29). Deltamethrin resistance followed the same pattern where IGF consumption increased deltamethrin-induced mortality from 3–11 days of age (1-way ANOVA: p<0.01, F = 26.2, df = 2). For TGF consumption, deltamethrin-induced mortality was increased at 3 days (2-sample t-test: p = 0.03; t = -2.31), 7 days (2-sample t-test: p<0.01; t = -8.31) and 11 days of age (2-sample t-test: p = 0.024; t = 2.43).

Malathion and λ-cyhalothrin resistance was not as strongly affected by the consumption of IGF and TGF, and no difference was observed in insecticide-induced mortality after the age of 3 days for λ-cyhalothrin when IGF was consumed (2-sample t-test: 7 days: p = 0.51, t = 0.67; 11 days: p = 0.5, t = 0.69; 15 days: p = 0.37, t = -0.92) or when TGF was consumed (2-sample t-test: 7 days: p = 0.81, t = -0.24; 11 days: p = 0.81, t = 0.24; 15 days: p = 0.33, t = 1.11). Similarly, there were no significant changes in malathion-induced mortality after the consumption of either IGF or TGF at the age of 7 days (2-sample t-test: p = 0.18; t = 1.87) or 15 days (2-sample t-test: p = 0.98; t = 0.03). There was, however, a significant increase in mortality at the age of 11 days after IGF consumption (2-sample t-test: p = 0.02; t = 2.44).

### The effect of exogenous dietary stressants on detoxification enzyme activity

**Fig 5:**
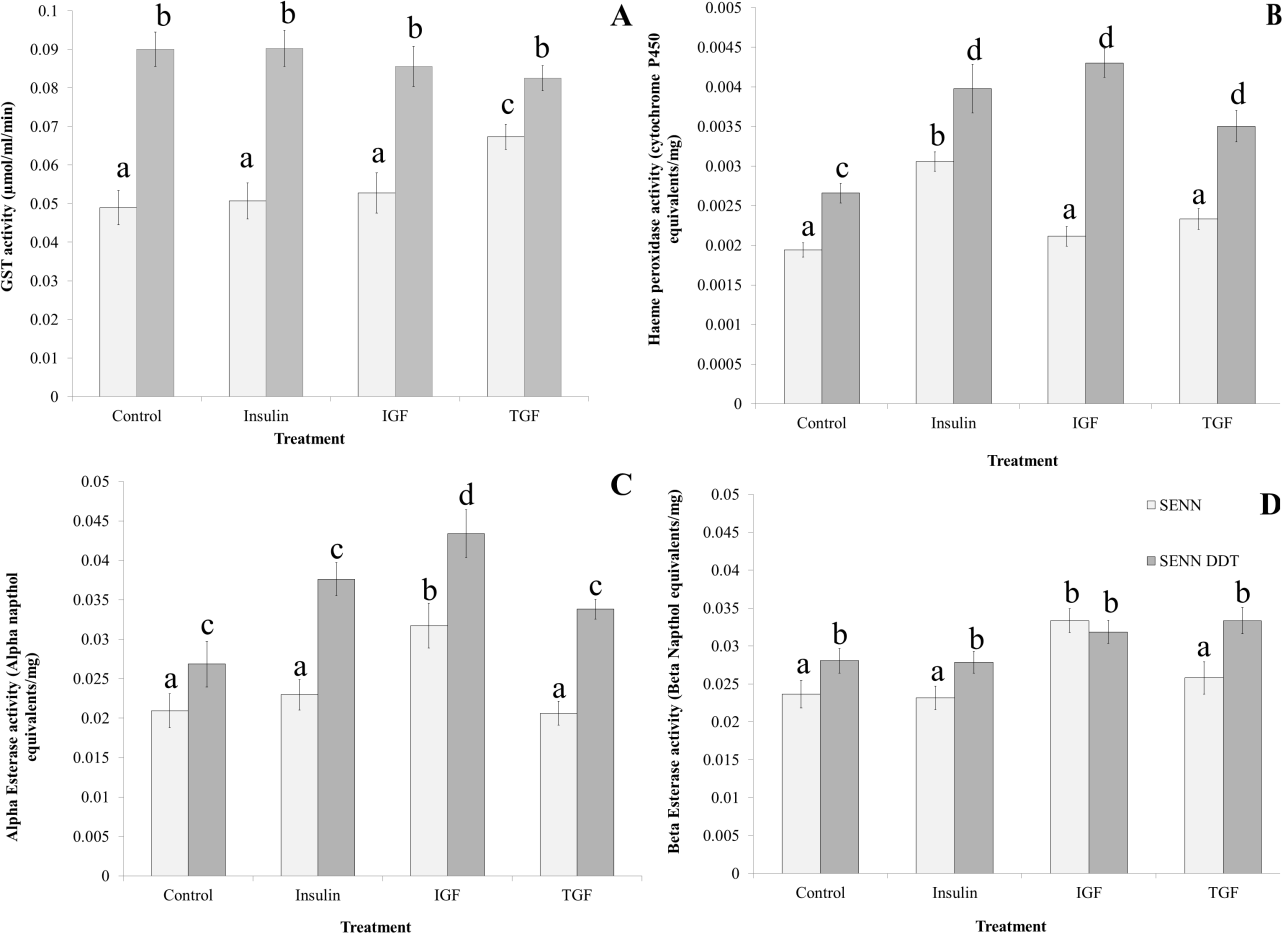
The effect of dietary supplementation on detoxification enzyme activity. A: Dietary supplementation did not cause any significant changes in SENN DDT GST activity. TGF significantly increased SENN GST activity, but no other treatment had a significant effect on GST activity in this strain. B: Insulin significantly increased cytochrome P450 activity in SENN, while no other treatment had a significant effect. For the SENN DDT strain, all treatments significantly increased P450 activity, but there were no significant differences between the treatments. C: IGF treatment was the only factor that significantly increased α-esterase activity in both strains. D: IGF treatment significantly increased β-esterase activity in SENN and SENN DDT, such that there was no significant difference between the strains.

The consumption of insulin, IGF and TGF had a variable effect on the activity of metabolic detoxification enzymes (Fig 5) in both the SENN and SENN DDT strains. GST activity in SENN DDT was not significantly affected by dietary supplementation (1-way ANOVA: p = 0.56; F = 0.68; df = 3). TGF consumption significantly increased GST activity in SENN (2-sample t-test: p<0.01; t = -3.69; df = 44), while none of the other treatments had any affect (1-way ANOVA: p = 0.32; F = 1.18; df = 2) (Fig 5A).

Cytochrome P450 activity, expressed in this experiment as haeme peroxidase activity, was affected by the consumption of exogenous dietary stressants, and there was a marked difference between strains (Fig 5B). SENN DDT females showed significantly higher levels of P450 activity following ingestion of insulin, IGF and TGF (1-way ANOVA: p<0.01; F = 21.6; df = 1). For the SENN strain, only insulin consumption significantly increased P450 activity (1-way ANOVA: p<0.001; F = 54; df = 1), while IGF and TGF consumption did not result in a significant change in P450 activity in this strain (1-way ANOVA: p = 0,06; F = 2.85; df = 2). In the SENN DDT strain, all dietary treatments resulted in a significant increase in P450 activity (1-way ANOVA: p<0.01; F = 11.2; df = 3). Insulin, IGF and TGF resulted in an increase in P450 activity that did not differ significantly from each other (1-way ANOVA: p = 0.06; F = 2.90; df = 2).

IGF was the only dietary stressant that affected α- and β-esterase activity in the SENN strain (2-sample t-test: α: p<0.01, t = -3.16; β: p<0., t = 4.72), while the other stressants did not result in a significant change in α-esterase (1-way ANOVA: p = 0.59; F = 0.53; df = 2) (Fig 5C) or β-esterase activity (1-way ANOVA: p = 0.44; F = 0.83; df = 2) (Fig 5D).

Dietary stressants had a variable effect on the general esterase activity in the SENN DDT strain. Similar to SENN, only IGF resulted in a significant increase in α-esterase activity (2-sample t-test: p = 0.01, t = -2.57) (Fig 5C). There was no significant difference in β-esterase activity in SENN DDT regardless of treatment (1-way ANOVA: p = 0.09; F = 2,21; df = 3) and these treatments did not differ from the increased β-esterase activity induced by IGF in the SENN strain (1-way ANOVA: p = 0.06; F = 2.36; df = 4) (Fig 5D).

### The effect of exogenous dietary stressants on oxidative stress defence enzyme activity

**Fig 6:**
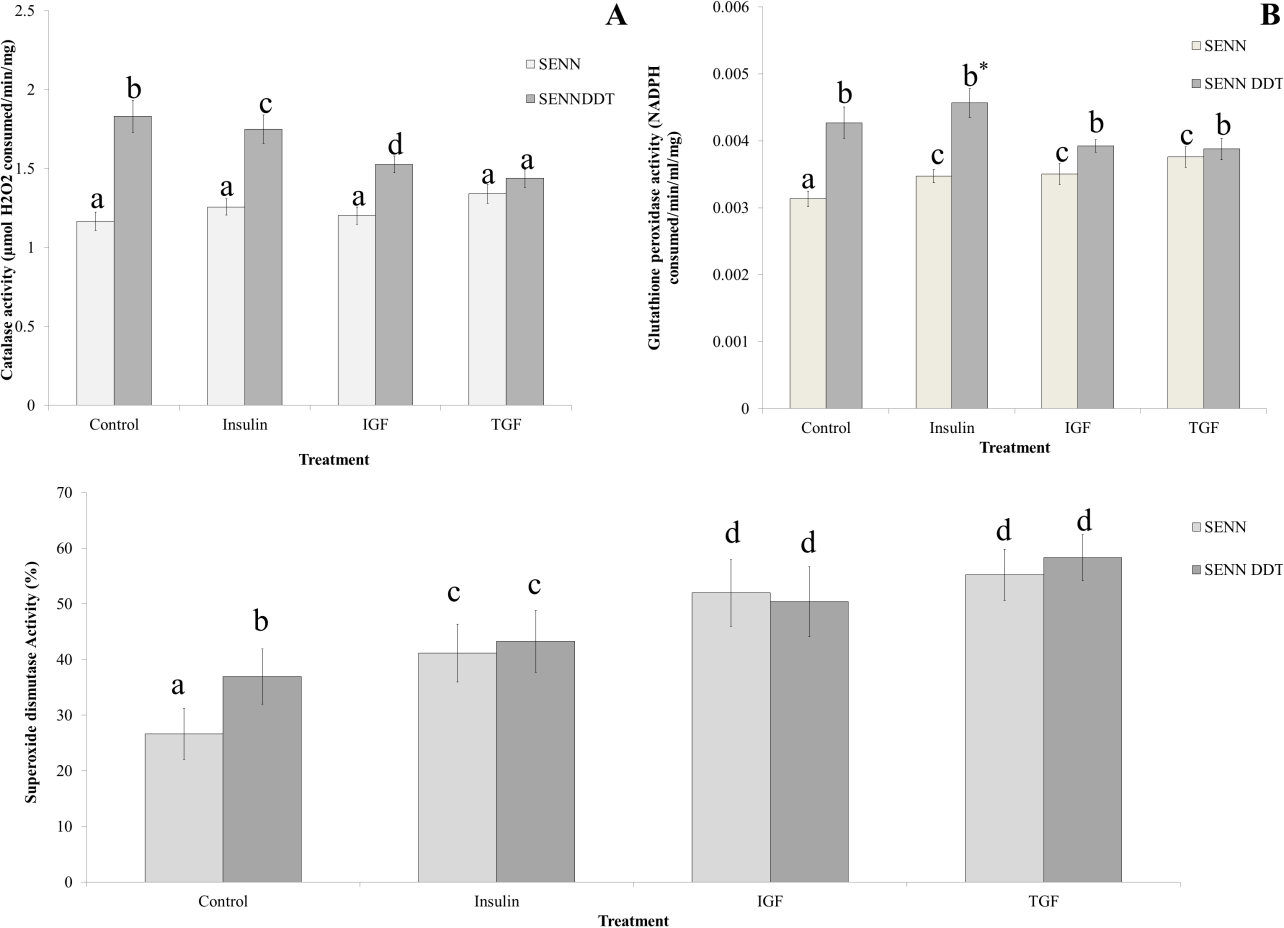
The effect of dietary supplementation on oxidative stress defence enzyme activity. A: Dietary treatments had no effect on the catalase activity of SENN, but significantly reduced catalase activity in SENN DDT, such that there was no significant difference in activity between the stains after TGF treatment. B: Glutathione peroxidase activity did not vary from the control after any treatment for either strain. For SENN DDT, insulin treatment resulted in the highest peroxidase activity compared to the other treatments. C: All treatments resulted in a significant increase in superoxide dismutase activity, such that there was no difference between strains by treatment.

None of the dietary treatments affected the levels of catalase activity in SENN (1-way ANOVA: p = 0.17; F = 1.73; df = 3). All dietary treatments significantly reduced catalase activity in SENN DDT (1-way ANOVA: p<0.01; F = 5.41; df = 3). Although catalase activity in SENN DDT was higher than SENN under control conditions (1-way ANOVA: p<0.01; F = 12.0; df = 3), the catalase activity in SENN DDT was reduced after TGF consumption such that it did not differ from catalase activity in SENN after TGF consumption (2-sample t-test: p = 0.34, t = 0.96) (Fig 6A).

In the SENN strain, dietary treatment significantly increased glutathione peroxidase activity (1-way ANOVA: p = 0.02; F = 3.50; df = 3), and the dietary treatments that increased the peroxidase activity by the same amount as the activity induced after the consumption of all three treatments do not differ from each other (1-way ANOVA: p = 0.33; F = 1.12; df = 2). For the SENN DDT strain, although none of the dietary treatments increased the glutathione peroxidase activity to higher than that of the control, insulin consumption resulted in the highest peroxidase activity of the 3 treatments (1-way ANOVA: p = 0.005; F = 5.61; df = 2) (Fig 6B).

Catalase activity was increased by dietary supplementation in both strains (1-way ANOVA: SENN: p<0.01, F = 38.1, df = 3; SENN DDT: p<0.01, F = 18.1, df = 3) (Fig 6C). There was no significant difference between strains for the each individual treatment (2-sample t-test: Insulin: p = 0.5, t = -0.67; IGF: p = 0.664, t = -0.44; TGF: p = 0.23, t = 1.23).

## Discussion

The supplementation of human host factors, whether hormonal such as insulin, or a growth factor such as IGF or TGF, has the capacity to affect longevity in *An*. *arabiensis*, a life history characteristic of epidemiological importance.

A previous study demonstrated that insulin supplementation via sucrose has a comparable effect on longevity as supplementation via an artificial blood meal [9]. This study builds on this work by including a few new variables. Firstly, these data suggest that changes in longevity induced in the insecticide susceptible SENN strain are due to hormonal activity, as denatured insulin does not induce the same effect. This change was not observed in the insecticide resistant strain SENN DDT. As it has been demonstrated previously that human insulin stimulates hydrogen peroxide production in the mosquito, this can possibly be attributed to SENN DDT’s increased capacity to cope with oxidative stress [16]. Although the resistance status of the strain used in the Kang *et al*. study [9] is not described, these results suggest that resistant and susceptible strains may respond differently to host factor ingestion.

Kang *et al*. [9] also demonstrated that consumption of the SOD mimetic MnTBAP negated the reduction in longevity in *An*. *stephensi*. While consumption of the mimetic had no effect on the SENN DDT strain, MnTBAP as well as MnTBAP and insulin reduced SENN longevity compared to the control. This may possibly represent a toxic effect of the mimetic on the SENN strain, which does not occur in the resistant SENN DDT strain. These data add to the body of research demonstrating the variable effects of oxidative stress and oxidative stress enzyme mimetics on various invertebrates [33, 34].

The consumption of low dose TGF did not significantly alter the longevity of either strain. Low dose IGF consumption, however, did cause a reduction in longevity compared to control females and TGF-treated females. A previous study demonstrated that IGF-fed *An*. *stephensi* increased reactive oxygen species levels in the midgut [25]. Therefore, the reduction in SENN longevity is consistent with the lower oxidative stress defence capacity of the SENN strain. In line with the findings of Drexler *et al*. [10], high dose IGF did not elicit any change in longevity in either strain, and neither did high dose TGF. SENN females treated with TGF did, however, live significantly longer than those treated with IGF. This was true for both the high and the low dosages.

The effect of host factor consumption on insecticide resistance phenotype has never been reported before. These data show that insulin consumption can modulate insecticide resistance phenotypes due to its’ hormonal effects, as denatured insulin did not cause a significant change in resistance phenotype. It should also be noted that the reduction in insecticide-induced mortality observed after insulin consumption was usually observed in younger mosquitoes i.e. under the age of 15 days. For the permethrin and deltamethrin resistance phenotypes, insulin consumption in mosquitoes 15 days and older can have a deleterious effect, with increased insecticide-induced mortality. This effect was more marked in malathion, where the increase in mortality was observed at the age of 7 days. From the age of 11 days, malathion resistance was almost completely negated, consistent with the phenotype observed in the SENN DDT strain in a previous study [15]. It is possible that the amelioration of the observed effects of age may be related to the digestion of the protein factors. Furthermore, it is interesting to note that TGF had an effect despite not undergoing the blood meal-induced gut activation it would under normal circumstances [11].

The effect of growth factor consumption on resistance phenotype was different to that of insulin. While insulin had a protective effect on younger mosquitoes, IGF and TGF consumption typically increased insecticide-induced mortality. DDT-induced mortality was increased at the ages of 3, 7, 11 and 15 days after IGF consumption. At the age of 3 days, deltamethrin, λ-cyhalothrin and malathion induced mortality was increased after IGF consumption, while DDT and pyrethroid-induced mortality was increased after TGF consumption. IGF consumption also consistently increased deltamethrin, but not λ-cyhalothrin, induced mortality. TGF consumption had no effect on malathion induced mortality, but increased deltamethrin-induced mortality up to the age of 11 days. Although these data does not provide a comprehensive answer as to why the protective effect of insulin consumption wears off with the time, the fact that ageing ultimately results in an increase in insecticide susceptibility in all treatmentsis probably an effect of mosquito senescence. For example, even though multiple blood meals maintain insecticide resistance levels for longer than in samples in whichif no blood is consumed, insecticide susceptibility still increases in the ageing mosquitoes [15]. The fact that any protective effects of insulin decaydecays in the older mosquito suggests that the effects of mosquito senenscence outweigh any protective effects gained from insulin consumption.

Of the xenobiotic detoxification enzymes, esterase activity was least affected by dietary supplementation. No changes were observed for β-esterases in SENN DDT, while the β-esterase activity in SENN was elevated to that of SENN DDT by IGF consumption. Similarly, α-esterase activity in both strains was significantly increased by IGF consumption. Dietary supplementation increased cytochrome P450 in SENN DDT, while only insulin increased P450 activity in SENN. Dietary supplementation suppressed GST activity in both strains to such an extent that the difference in GST activity observed between the strains under control conditions was negated.

Glutathione peroxidase activity was not affected by dietary supplementation. Superoxide dismutase activity was higher in the SENN DDT strain under control conditions, and although host factor consumption increased SOD activity, it did so equally in both strains. Catalase activity in SENN was unaffected by dietary consumption, while it was significantly reduced in the SENN DDT strain.

It is concluded that the consumption of host factors including insulin, IGF and TGF can effect vector mosquitoes, corroborating previous research by Kang *et al*. [9], Drexler *et al*. [10] and as reviewed by Pakpour *et al*. [11]. Although the experimental protocol adopted here supplied these supplements via sucrose as opposed to blood ingestion it does highlight several important factors. The first, and possibly most important, is that insecticide resistance phenotypes in adult females are affected by ingestion of these factors and that, conversely, insecticide resistance affects the physiological response to the ingestion of these factors. Secondly, it is suggested that the consumption of blood during the lifetime of adult females will induce complex pleiotropic effects on their life histories over and above their reproductive capacities. This study also adds to the body of data about the interplay between oxidative stress and insecticide resistance. Previous studies highlighted that oxidative stress defence plays a direct role in the insecticide resistance [16] and that biological functions, such as blood feeding, that modulate oxidative stress levels can affect not only insecticide resistance but longevity as well [15]. This study suggests that not only blood itself, but the host factors in the blood contribute to the effect of to multiple blood meals. Furthermore, as insulin, IGF and TGF all induce oxidative stress in various insects [9,11,25] this highlights the crucial role of oxidative stress in the biologyof *Anopheles* mosquitoes.
